# Corneal Lenticule Creation Using a New Solid-State Femtosecond Laser Measured by Spectral Domain OCT in a Porcine Eye Model

**DOI:** 10.1167/tvst.11.6.20

**Published:** 2022-06-22

**Authors:** Christoph Lwowski, Anna Voigt, Karel Van Keer, Thomas Kohnen

**Affiliations:** 1 Department of Ophthalmology, Goethe-University Frankfurt am Main, Frankfurt am Main, Germany; 2 Department of Ophthalmology, University Hospitals Leuven, Leuven, Belgium

**Keywords:** lenticule extraction, femtosecond laser, SmartSight, corneal refractive surgery

## Abstract

**Purpose:**

To determine the accuracy and precision of corneal lenticule creation with a new solid-state femtosecond laser in a porcine eye model.

**Methods:**

Corneal lenticule creation was performed using a new solid-state femtosecond laser on 60 porcine eyes with 10 subgroups. Optical coherence tomography images were acquired immediately after laser treatment. Cap thickness (CT), cap diameter (CD), and lenticule thickness (LT) were measured manually by three independent readers. Additionally, CT and LT were measured by an automated algorithm (aLT, aCT).

**Results:**

Measured LT was significantly greater than the intended LT (average difference 14.3 ± 5.6 µm, *P* < 0.001). aLT was closer but still significantly different from the intended LT (−2.9 ± 5.8 µm, *P* < 0.001). Measured CT showed no significant difference from the intended CT (2.6 ± 13.3, *P* = 0.145). aCT was significantly smaller compared to the intended CT (−9.6 ± 13.6, *P* < 0.001). Measured CD was significantly smaller compared to the intended CD (−0.21 ± 0.20 mm, *P* < 0.001). All lenticules were cut as planned with no laser-related complications.

**Conclusions:**

This new solid-state femtosecond laser used in our trial provides corneal lenticule creation in a porcine eye model comparable to other established systems. However, measuring those lenticules in the provided setting seems too challenging even when using semiautomated algorithms, which seems to be due to the experimental setting of the trial.

**Translational Relevance:**

This trial shows the precision and repeatability of corneal cuts performed by a new femtosecond laser that could translate to refractive corneal lenticule surgery.

## Introduction

With an increasing demand of being spectacle independent due to modern aesthetics and active lifestyle, and myopia becoming more common in the Western world,^1^ refractive surgery is becoming one of the most popular fields in recent ophthalmology. Since most of the patients who present themselves in our practice have a rather low to medium refractive error, laser treatment of the cornea is still the most common procedure worldwide.^2^^,^^3^ In particular, laser in situ keratomileusis, with its fast visual rehabilitation combined with high postoperative comfort and a low rate of complications, is currently the most used technique.^4^^–^^6^ Nevertheless, in the past 25 years, a new procedure, called lenticule extraction, has been introduced in which a lenticule is cut with a femtosecond laser and either taken out through a flap (femtosecond lenticule extraction [FLEX])^7^ or a cut (small incision lenticule extraction [SMILE]^8^ [Carl Zeiss Meditec, Jena, Germany] or corneal lenticule extraction for advanced refractive correction [CLEAR]^9^ [Ziemer, Biel, Switzerland]). Those procedures have become more popular because, especially in SMILE procedures, no flap-associated complications and prolonged dry eye symptoms occur.^10^^,^^11^ Simultaneously, the short- and long-term results seem to be comparable with the well-established femtosecond laser assisted laser in situ keratomileusis (fs-LASIK) treatment.^10^^,^^12^ Another factor in favor of lenticule extraction is that the surgeon does not need an excimer since all cuts are made by the femtosecond (fs) laser.

Now with SMILE being commercially used worldwide, other laser manufacturers such as Ziemer (CLEAR) and SCHWIND eye-tech-solutions (SmartSight) have started to introduce fs lasers and femtosecond profiles to offer surgeons a broad range of different machines and treatment patterns.

The SCHWIND ATOS laser system and the SmartSight treatment (both SCHWIND eye-tech-solutions GmbH, Kleinostheim, Germany) have had the Conformité Européenne (CE) mark since July 2020. First clinical results seem to indicate that the laser and the treatment are able to reach similar results compared to the established systems.^13^

Features of this solid-state femtosecond laser would be cyclotorsion correction before, during, and after docking; a low-dose treatment with typically less than 100 nJ per pulse; no minimum lenticule thickness; a progressive, refractive transition zone and therefore less undercorrection; less regression; and a better centration of the optical zone. The laser source is a sophisticated solid-state laser, and it works with a 1030 ± 50-nm wavelength to produce femtosecond pulses with a pulse duration of 225 ± 70fs.

To reach good clinical results, the corneal cuts need to be accurate, precise, and reproducible for different optical zones, refractive errors, and cap/lenticule thicknesses and diameters. Prior studies have shown that not only in LASIK but also in SMILE eyes, there can be a deviation of thickness and diameter of flap or lenticule, respectively, depending on laser type and intended depth.^14^^–^^16^ Therefore, this study was conducted to examine the accuracy and precision of the cap and lenticule thickness and diameter for the currently introduced SmartSight protocol using a new all solid-state femtosecond laser.

## Methods

### Femtosecond Laser

The ATOS Laser System (version 1.5.0.1; SCHWIND eye-tech-solutions) is a recently introduced solid-state femtosecond laser working at 1030 ± 50 nm. It offers eye tracking, cyclotorsion correction, and pupil recognition, which lead to efficient astigmatic corrections. The repetition rate is up to 4 MHz with typical treatment energy of less than 100 nJ (maximum laser output <500 mW) and pulse duration 225 ± 70 fs.

### SmartSight Protocol

**Figure 1.**
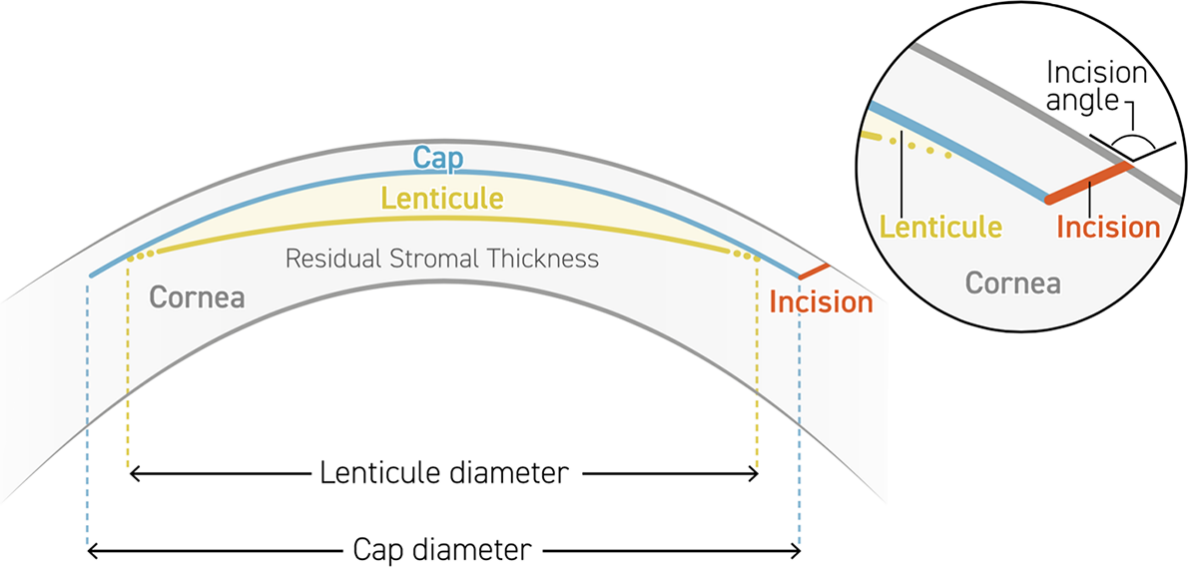
Schematic image of the SmartSight protocol.

**Figure 2.**
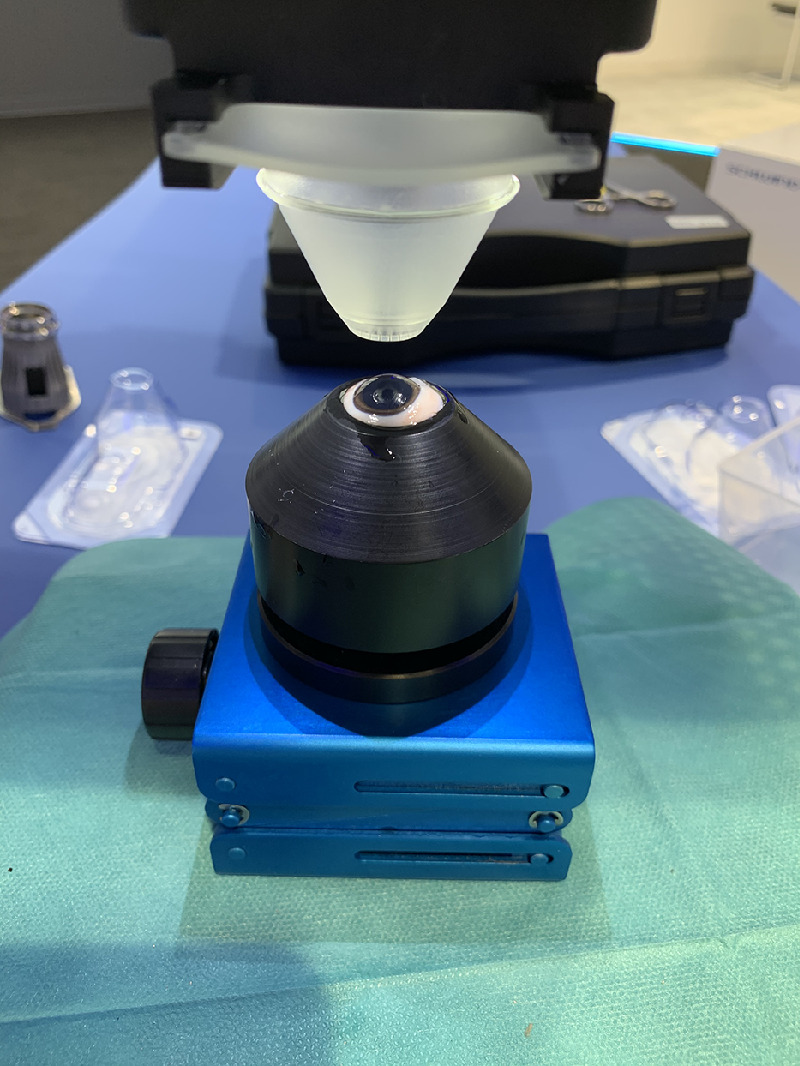
Porcine eye underneath the laser interface prior to docking.

The SmartSight protocol for the femtosecond laser is a lenticule-based protocol for corneal myopic correction. It first produces the refractive cut with a possible diameter of the optical zone from 5.5 to 7.5 mm extended by the required progressive, refractive transition zone, granting a standardized transition zone that automatically adjusts to the intended correction. Afterward, it produces the anterior cut with a diameter of 6.5 to 9.0 mm at a cap thickness selectable from 100 to 160 µm. The last step is the edge cut or incision with an angulation from 45° to 135° and an arc length from 2.0 to 5.0 mm (Figs. 1, 2).

### Experimental Protocol

Sixty freshly enucleated porcine cadaver eyes were kept in a wet container at +10°C and used within a maximum of 8 hours after enucleation. Immediately prior to surgery, the epithelium was manually removed and the eye was placed in a custom holder to ensure stabilization and adequate intraocular pressure. All treatments were performed by a single physician (CL), and attributes of the intended treatment are listed in Table 1 as entered in the laser software. Fixed parameters were set as follows:

1. Spot and track distances 3 µm for the lamellar cuts
2. 1.5 µm edge distance for the edge cuts
3. 120° edge cut angle
4. 100 nJ per pulse

**Table 1.** Refractive Treatment Characteristics

Setting	Sphere (Sph)	Cylinder (Cyl)	Axis	Optical Zone, mm
1	−10	0	0	6.0
2	−10	−5	0	6.0
3	−6	0	0	6.5
4	−6	−3	0	6.5
5	−3	0	0	7.0
6	−3	−1.5	0	7.0
7	−6	−3	0	5.5
8	−6	−3	0	6.0
9	−6	−3	0	7.0
10	−6	−3	0	7.5

Incision width: 3 mm.

Incision angle: 120°.

Cap thickness: 135 µm.

Cap diameter: 8.5 mm.

Ten different lenticule profiles were created with six eyes in each group to evaluate the laser for low, moderate, and high myopia and different amounts of astigmatism. The refractive characteristics can be found in Table 1.

### Optical Coherence Tomography Imaging

Immediately after laser treatment, the porcine eye was imaged using a high-resolution spectral domain optical coherence tomography (SD-OCT) (TELESTO SP5 Spectral Domain OCT, with 1325 nm at 91 kHz; software: ThorImage OCT 4.2; Thorlabs, Newton, New Jersey, USA). A 9-mm line scan was acquired over the 6- to 12-o'clock and 3- to 9-o'clock axis. All images were taken by the same ophthalmologist (KVK).

### Measurements

The OCT images were exported as grayscale .png files and imported into ImageJ (version 1.53a, Wayne Rasband; National Institutes of Health, Bethesda, MD, USA).

The following parameters were measured:

1. Central cap thickness (CT)
2. Cap diameter (CD)
3. Central lenticule thickness (LT)

The measured values for the LT had been compared with the entered values after those had been converted from human to porcine corneas based on “A Simple Cornea Deformation Model.”^17^ They are listed as the “intended” values in the following.

**Figure 3.**
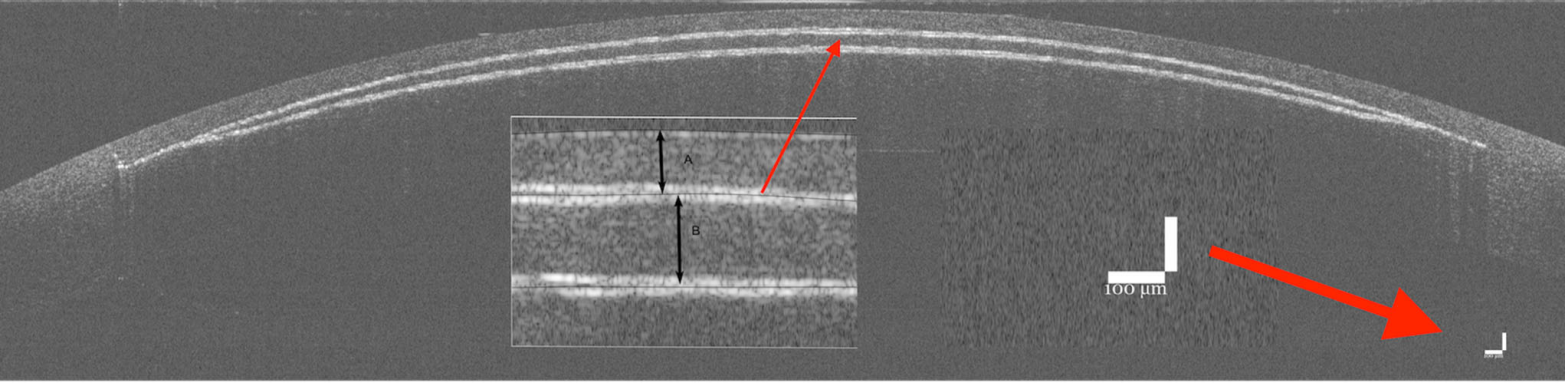
(a) OCT image of a treated cornea. (b) Closeup of the OCT image, including markers for the epithelium, cap, and lenticule cut. A = cap thickness; B = lenticule thickness.

All parameters were measured by three independent readers (CL, KVK, AV) for two (6- to 12-o'clock and 3- to 9-o'clock axis) images per porcine eye, and the mean values (of the six readings) were used. CT and LT were measured perpendicular to, and in the middle of, the virtual line connecting the peripheral boundaries of the cap incision (CD). We compared the mean values of the measurements to the intended parameters and evaluated the interobserver repeatability. The measurement scale was set accordingly to the pictured scale on the Thorlabs’ exports (Fig. 3).

In an effort to improve the accuracy of the measurements, the axial measurements (CT and LT) were measured using a semiautomated method (aCT and aLT, respectively). The raw .png image file was imported into Python (Python 3.9.10; https://www.python.org/) using the OpenCV library (https://opencv.org/). A line plot was drawn across the central axial line, summing 100 adjacent pixels (50 to either side of the central line) in the coronal plane. Local peaks in this curve were automatically detected using the scipy find_peaks module (SciPy 1.8.0; https://scipy.org/) and subsequently overlaid on the original image for user verification.

### Statistical Analysis

Data were analyzed using SPSS 24.0 for Mac (SPSS, Inc., Chicago, IL, USA). The measurements were averaged over the three readers. Normality of the distribution was evaluated using the Kolmogorov–Smirnov test. Continuous variables were summarized as mean ± standard deviation. The difference between intended versus achieved cuts was analyzed using a one-sample *t*-test for individual eyes and using one-way analysis of variance with Bonferroni post hoc testing to compare the differences between groups. The measurements of the independent observers were assessed for internal consistency using Cronbach's α.

Based on previous reports evaluating the intended versus achieved flap thickness in a similar setting after femtosecond flap creation in porcine eyes with an achieved flap thickness of 110 µm for an intended thickness of 120 µm,^18^ to achieve a power of 95% at an α of 0.05, a sample size of 15 eyes was needed.

Statistical significance was defined for *P* < 0.05 (prior to the Bonferroni correction).

## Results

The results of our experimental study using 60 porcine eyes divided into 10 subgroups with 6 eyes each were analyzed. The difference in intended measures and treated spherical equivalent (SE) between the groups can be found in Table 1. In Table 2, the values CT, CD, and LT correspond to the calculated values expected for porcine corneas as shown by Tobias Kehrer and Samuel Arba Mosquera.

**Table 2.** OCT Measurements

	Lenticule Thickness (µm)			Cap Thickness (µm)			Cap Diameter (mm)	
Setting	Intended	Manually Measured, Mean ± SD	Automated, Mean ± SD	Intended	Manually Measured, Mean ± SD	Automated, Mean ± SD	Intended	Measured, Mean ± SD^a^
1	125	146.5 ± 5.7	126 ± 7.7	126	152.0 ± 17.1	131.2 ± 18.6	7.9	7.8 ± 0.2
2	169	180.6 ± 6.8	165 ± 3.1	126	130.7 ± 11.9	118.8 ± 7.9	7.9	7.7 ± 0.4
3	100	111.8 ± 3.7	95.6 ± 4	126	127.9 ± 7.2	112.9 ± 6.9	7.9	7.6 ± 0.2
4	134	151.1 ± 6.2	132.6 ± 4.3	126	133.0 ± 12.9	123.1 ± 16.1	7.9	7.7 ± 0.1
5	65	80.2 ± 6.4	64.5 ± 11.3	126	130.5 ± 8.1	123.6 ± 12.6	7.9	7.6 ± 0.2
6	91	106.7 ± 3.6	87.8 ± 4.3	126	124.0 ± 7.8	113.9 ± 14.7	7.9	7.9 ± 0.4
7	99	113.8 ± 4.3	97.8 ± 5.1	126	118.3 ± 6.6	108.5 ± 8.7	7.9	7.7 ± 0.2
8	116	128.6 ± 3.3	111 ± 7.5	126	122.2 ± 8.2	111.1 ± 8.0	7.9	7.8 ± 0.7
9	153	166.6 ± 3.6	147 ± 4.1	126	120.3 ± 11.0	106.1 ± 11.0	7.9	7.9 ± 0.5
10	174	182.8 ± 3.8	168.9 ± 5.6	126	125.4 ± 11.3	114.1 ± 13.7	7.9	7.9 ± 0.2
All eyes	122.1 ± 33.6	136.4 ± 33.1*P* = 0.00	118.2 ± 33.4*P* = 0.00	126	128.6 ± 13.4*P* = 0.145	116.4 ± 14.0*P* = 0.00	7.9	7.8 ± 0.2*P* = 0.00
Mean difference from intended for all eyes		14.3 ± 5.6	−2.9 ± 5.8		2.6 ± 13.3	−9.6 ± 13.6		−0.2 ± 0.2

a Manual measurement as average of three observers of two scans per observer per eye.

**Figure 4.**
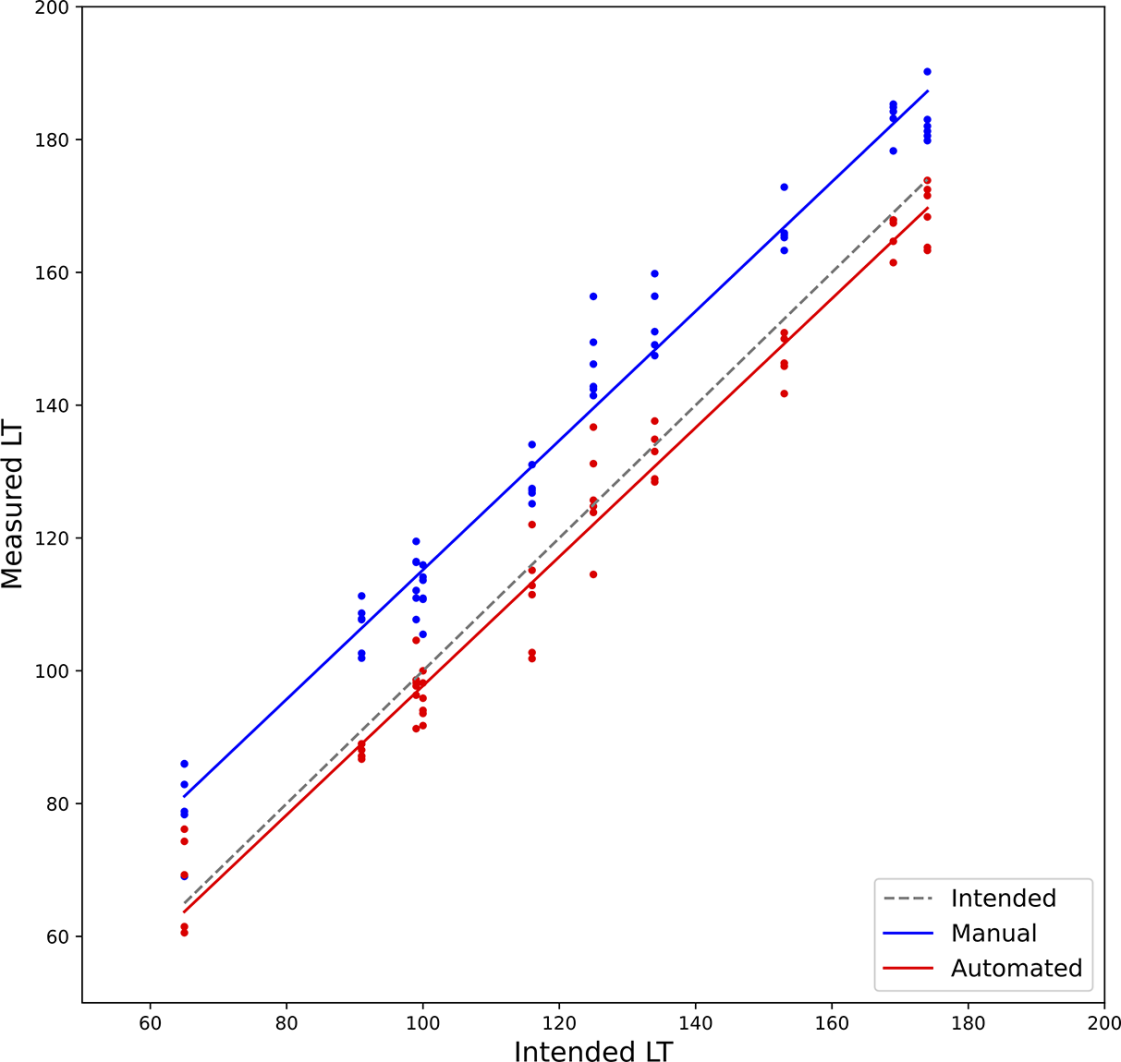
Correlation between intended and measured LT.

Overall, the measured LT (averaged over the three observers) was significantly larger than the intended LT (average difference 14.3 ± 5.6 µm, *P* < 0.001; Fig. 4). Measured and intended LT showed a strong linear correlation (*R*^2^ = 0.97, *P* < 0.001, Fig. 4). Measured CD was significantly smaller compared to the intended CD (−0.2 ± 0.2 mm, *P* < 0.001). The only group not having a significant difference between measured and intended CD was group 1 (7.80 ± 0.20 mm). In all other groups, the deviation was significantly different from 0 (one-sample *t*-test, *P* < 0.001). The lowest mean deviation was found in group 10 and the highest in group 3 (7.9 ± 0.2 mm vs. 7.6 ± 0.2 mm; *P* < 0.001). However since the sample size per group is only six eyes, this finding does not necessarily have to be relevant.

**Figure 5.**
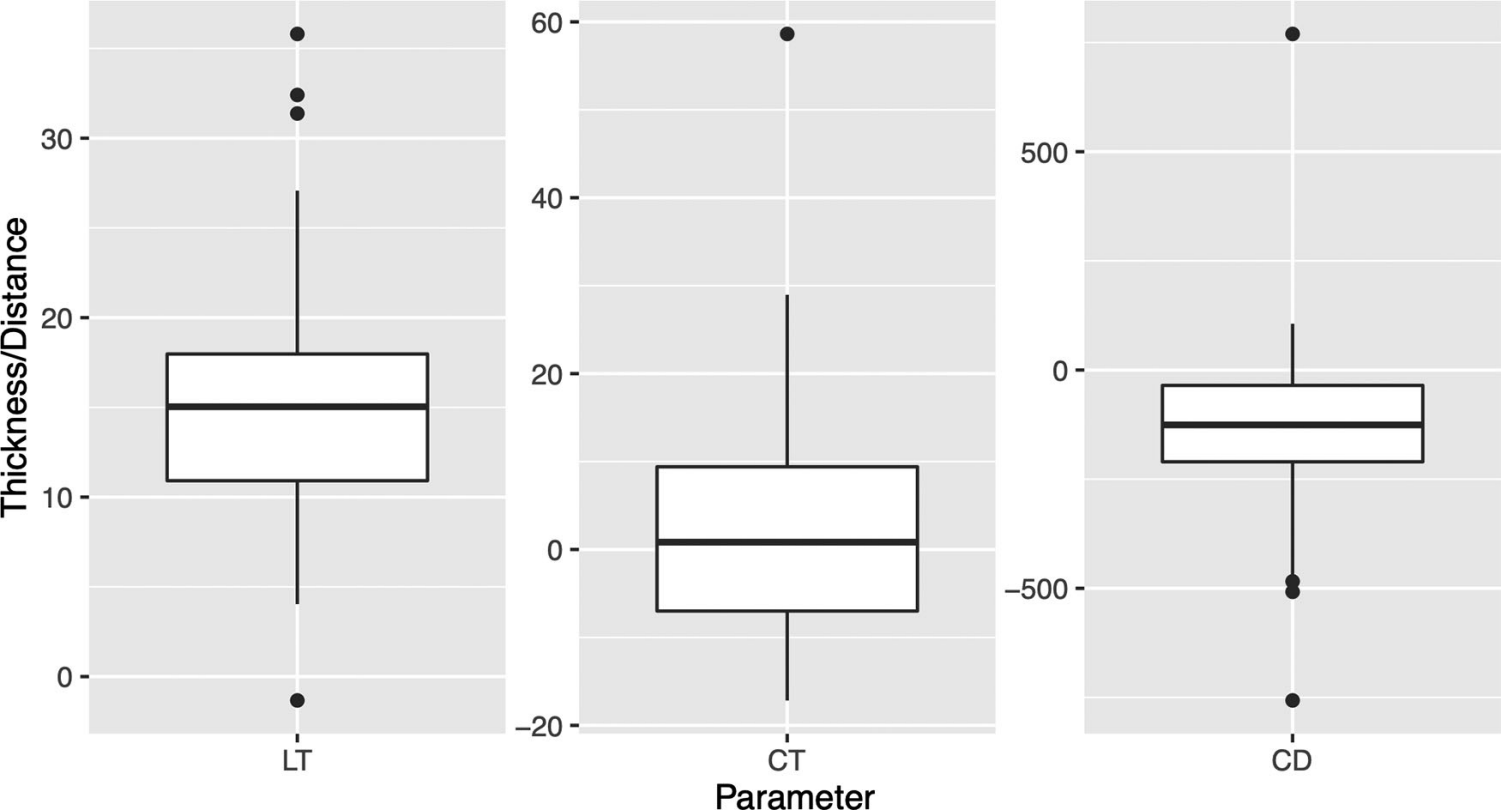
Deviation from calculated LT, CT, and CD.

Measured CT showed overall no significant difference from the intended CT (average difference 2.6 ± 13.3 µm, *P* = 0.145, Fig. 5) but with a higher SD compared to LT.

The difference between measured and intended LT showed significant differences between the different treatment groups (*P* = 0.007, Fig. 5), with a significantly higher difference in group 1 (highest difference at 21.5 ± 5.2 µm) compared to group 10 (lowest difference at 8.8 ± 3.5 µm) (*P* = 0.002), but no other significant differences between groups. For CT, group 1 showed significantly higher differences compared to groups 3 and 5 to 11. CD showed no significant difference between groups concerning the difference between intended and measured diameter (*P* = 0.505).

**Figure 6.**
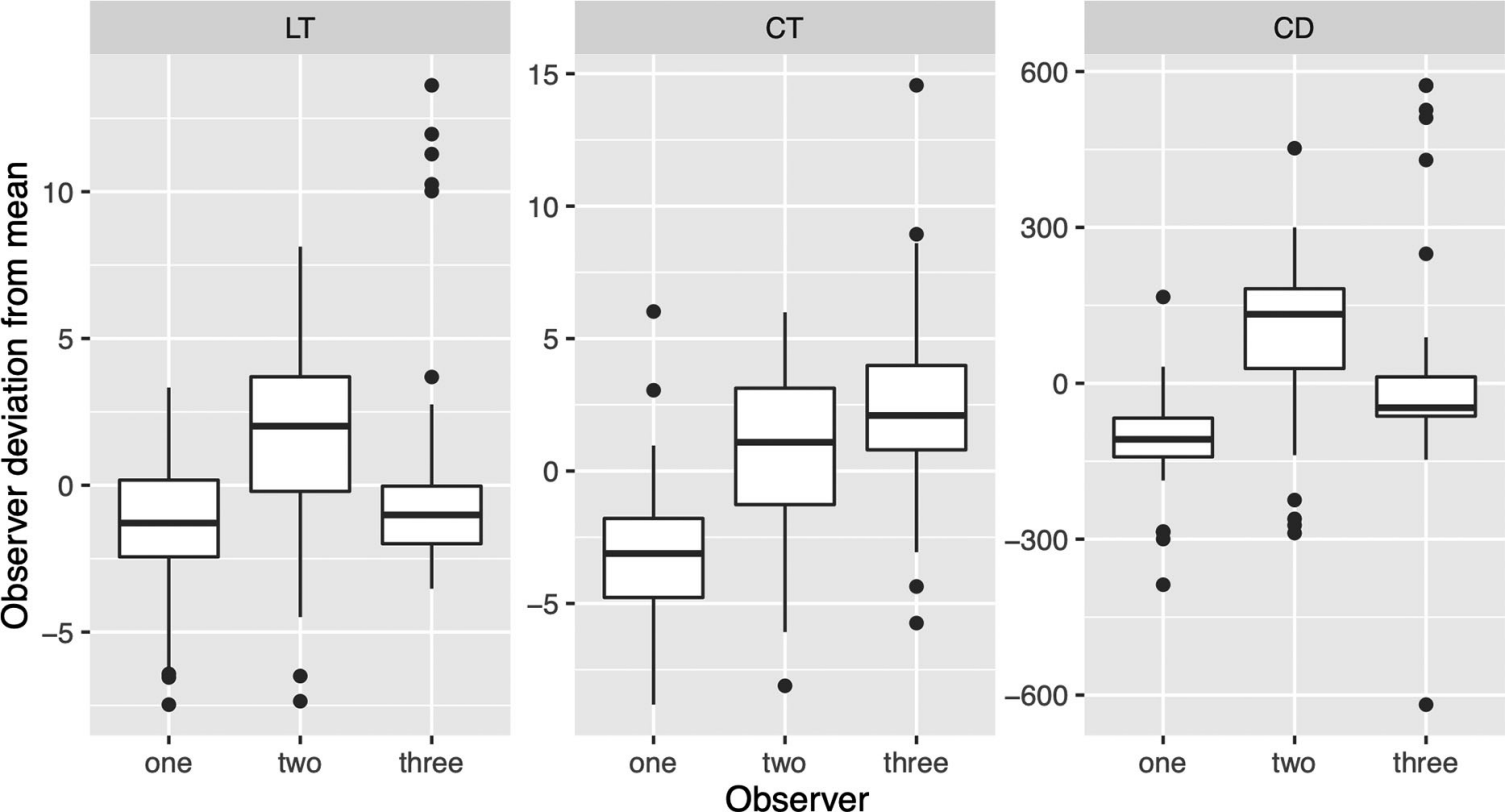
Graph depicting the difference of the individual observers with the overall average.

Interobserver repeatability was high in all groups for all measures with a Cronbach's α of 0.998, 0.980, and 0.852 for LT, CT, and CD. The average interobserver differences are depicted in Figure 6.

The variability of the manual measurements was assessed by analyzing the intraclass correlation coefficients (ICCs) of the six individual measurements before averaging. For LT, CT, and CD, the ICCs were 0.988, 0.902, and 0.541, respectively.

**Figure 7.**
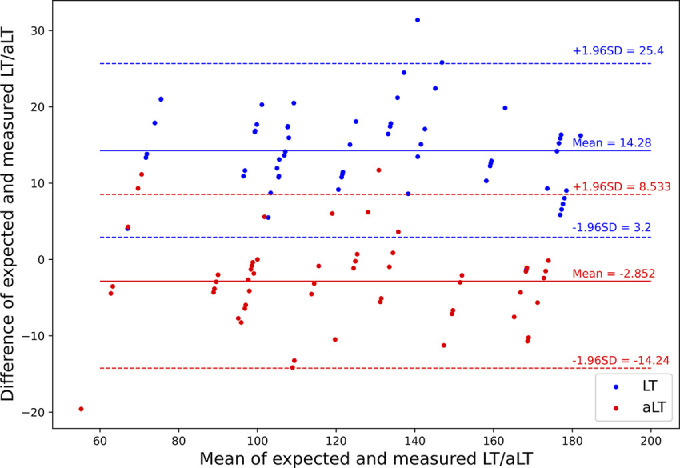
Bland–Altman plot comparing intended versus measured LT and aLT.

**Figure 8.**
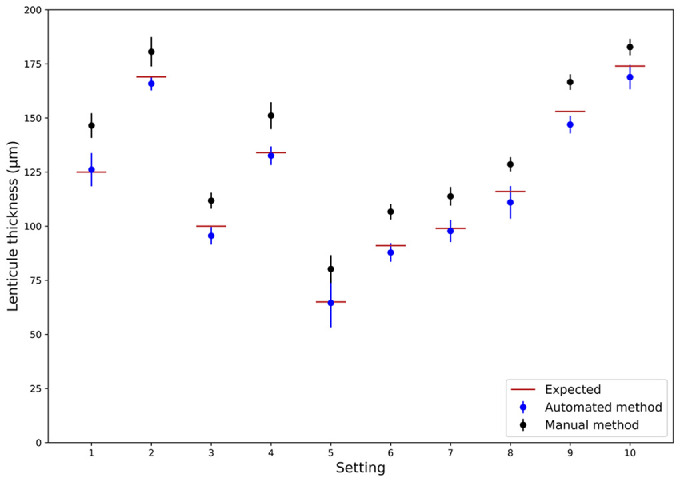
Graph depicting the expected manually and automatically measured lenticule thickness.

Using the automated measurement, the average measured lenticule thickness (aLT) was closer to the intended LT compared to the manual method but had a higher standard deviation (−2.9 ± 5.8 µm for the automated vs. 14.3 ± 5.6 µm for the manual method) (Table 2, Figs. 7, 8).

The absolute error and standard deviation of aCT were larger than the manual measurements (−9.6 ± 13.6 µm vs. 2.6 ± 13.3 µm).

## Discussion

We present the results of an experimental study using 60 porcine eyes in terms of precision of lenticule and cap measurements for different treatment groups using a new treatment protocol for myopic correction with a recently introduced solid-state femtosecond laser system from SCHWIND eye-tech-solutions GmbH. Since the SmartSight procedure is new to the market, we cannot compare it to other studies that used the same laser and protocol but rather compare it to studies with other lasers and/or treatment protocols or to femtosecond LASIK studies. Using porcine eyes, we had to use a correction for the difference in corneal curvature according to the deformation model of Kehrer and Mosquera as mentioned above, ex vivo setting, and morphology of the eyes themselves. Therefore, the deviation could be smaller in human eyes in an in vivo setting. Additionally, the measurements were taken by individual observers, and the lenticule center was estimated by using the connection line between the cap edges. However, we tried to compensate for this bias by using the mean of three observers who measured two images per eye.

The intended CT was the same for all eyes and showed no significant overall deviation in any group except for group 1. Similar results with excellent agreement of intended and measured cap thickness are shown by Reinstein et al.^19^ and Zhao et al.^20^ using the Visumax laser platform (Carl Zeiss Meditec) or for LASIK flaps with a deviation of less than 10 µm in eye bank corneas,^15^ but some of those studies show a similar deviation but with a lower SD than in our data. A study by Colombo-Barboza was able to show a higher deviation from the intended measures for LASIK flaps but was significant at only 2 of 20 points measured,^21^ and Parafita-Fernandez et al.^22^ had significantly thicker flaps than intended. Alio Del Barrio et al.^23^ found a thicker cap than expected created by the Visumax laser platform (Carl Zeiss Meditec), in contrast to fs flaps that were closer to the target thickness in the same trial, but Ozgurhan et al.^24^ reported that they did not find a significant deviation from intended cap thickness for either SMILE or fs-LASIK. Interestingly, the automated measures (aCT) found a higher deviation from the intended CT. One possible explanation could be the difficulty to clearly delineate the corneal epithelium surface in cases where a hyperreflective mirror artifact coalesces with the corneal surface.

Additionally, it remains to discuss if the initially higher deviation of measurements in group 1 is due to a learning curve in the first treated and measured eyes, which is reported as well by Asena and Donmez,^25^ who report a learning curve in terms of flap thickness in fs-LASIK for their first 20 eyes of the trial. Still, one has to discuss if a deviation from intended measures is clinically relevant as long as there is no downside to the precision in terms of treated SE. Nevertheless, CT and LT are essential in terms of corneal integrity and biomechanical properties.^26^ The cap diameter was significantly smaller compared to the intended diameter but without a relevant variability in between the groups. Other works, such as Li et al.,^27^ reported a larger optical zone than expected in SMILE eyes using the Carl Zeiss Meditec Visumax platform, and Damgaard et al.^16^ reported similar results in comparison to fs-LASIK, but as we had a different and experimental setting, this is not completely comparable to our measures. With a deviation of 2% CD (–0.2 ± 0.2 mm) from the target diameter, the tested laser setting does show a high accuracy.

However, the lenticule diameter is as important as the cap diameter but was intentionally not measured since it would be much harder to define in the OCT images and therefore the measurements would be not as valid. This is especially due to the transition zone. Therefore, we cannot make a conclusion on the optical zone diameter. Since we are reporting the first results of this highly experimental setting with ex vivo measurement of porcine eyes after lenticule creation, we did not include further parameters such as LT at different positions and calculation of the refractive power of the lenticule. Nevertheless, this would be an interesting addition to measurements in further study protocols.

Regarding the LT, the deviation from the intended thickness was significant for all eyes with a thicker lenticules than calculated. The difference was highest in group 1 and lowest in group 8. This seems to go hand in hand with trials on ablation depth, overall CT reduction, or LT, which show a higher ablation than expected.^28^^–^^30^ A recently published trial by Luo et al.^31^ showed significant deviation from targeted stromal reduction in SMILE eyes but fs-LASIK as well. In the SMILE group, the stromal reduction was overestimated by 20.05 ± 5.92 µm and overestimated by 8.21 ± 8.14 µm in fs-LASIK eyes. However, they report that this discrepancy was not associated with over- or undercorrection. Alio Del Barrio et al.^32^ also reported a significant deviation from stromal reduction in a recent study for SMILE eyes during a 6-month follow-up (–13.21 ± 7 µm). Nevertheless, one has to keep in mind that ablation depth in LASIK is performed by an excimer and not an fs laser. However, from the manufacturer's side, it was already expected to have a thicker lenticule, and SCHWIND developed in the meantime a software update with an enhanced precision in terms of this. Compared to the manually measured LT, the aLT measurements had a much improved absolute agreement with intended LT. From the authors’ point of view, this could be due to the two bubble layers that define the lenticule and make it hard to measure them manually. Because these bubble layers fluctuate over the course of the cuts, the exact positioning of the measurement affects the results and thus explain why the standard deviation remains rather high, even in the automated method.

Limitations of the reported study are that porcine eyes offer limited conclusions on human eyes, but the precision should be similar. The same does account for ex vivo eyes. Additionally, we only report anatomic parameters and offer no conclusion on optical quality, SE, stability, or optical zone. A possibly small inaccuracy of the OCT measurements themselves needs to be taken into account as well as the fact that the measurements were not acquired using the provided Thorlabs’ software. Not correcting for variations in refractive index and optical aberrations could theoretically negatively influence the accuracy of the results when measuring distances in a third-party application, especially for measurements along the CD.

The measured thicker value for LT compared to the entered/expected value is explainable through approximately 1 diopter of overcorrection due to the differences in design from ex vivo porcine to in vivo human corneal response (e.g., different curvatures, refractive index, biomechanics). This can be inferred from the fact that the measured cap thickness was as planned.

Still, existing studies on ex vivo porcine eyes suggested these are a sufficient replacements for living corneas in laser safety trials.^33^

With 60 eyes for the mean parameter of cap thickness, the study power is high enough to discover relevant differences, but reading the results of the subgroups, one has to take the smaller group size into account.

## Conclusion

This new solid-state femtosecond laser system using a currently introduced lenticule-based corneal refractive surgery protocol offers a precise and accurate cap thickness with deviations in cap diameter and lenticule thickness that are comparable to established systems and treatment protocols. However, measuring porcine eyes ex vivo seems to be challenging, which seems to be due to the experimental setting, and needs further improvement.
